# Macrophage scavenger receptors: Tumor support and tumor inhibition

**DOI:** 10.3389/fonc.2022.1096897

**Published:** 2023-01-06

**Authors:** Elena Kazakova, Pavel Iamshchikov, Irina Larionova, Julia Kzhyshkowska

**Affiliations:** ^1^ Laboratory of translational cellular and molecular biomedicine, National Research Tomsk State University, Tomsk, Russia; ^2^ Cancer Research Institute, Tomsk National Research Medical Center, Russian Academy of Sciences, Tomsk, Russia; ^3^ Laboratory of Genetic Technologies, Siberian State Medical University, Tomsk, Russia; ^4^ Institute of Transfusion Medicine and Immunology, Mannheim Institute for Innate Immunoscience (MI3), Medical Faculty Mannheim, University of Heidelberg, Mannheim, Germany; ^5^ German Red Cross Blood Service Baden-Württemberg – Hessen, Mannheim, Germany

**Keywords:** tumor-associated macrophage, scavenger receptor, angiogenesis, extracellular matrix, cancer, tumor microenvironment, endocytosis, phagocytosis

## Abstract

Tumor-associated macrophages (TAMs) are a heterogeneous population of myeloid cells that constitute up to 50% of the cell mass of human tumors. TAMs interact with the components of the tumor microenvironment (TME) by using scavenger receptors (SRs), a large superfamily of multifunctional receptors that recognize, internalize and transport to the endosomal/lysosomal pathway apoptotic cells, cytokines, matrix molecules, lipid modified lipoproteins and other unwanted-self ligands. In our review, we summarized state-of-the art for the role of macrophage scavenger receptors in tumor development and their significance as cancer biomarkers. In this review we focused on functional activity of TAM-expressing SRs in animal models and in patients, and summarized the data for different human cancer types about the prognostic significance of TAM-expressed SRs. We discussed the role of SRs in the regulation of cancer cell biology, cell-cell and cell-matrix interaction in TME, immune status in TME, angiogenesis, and intratumoral metabolism. Targeting of tumor-promoting SRs can be a promising therapeutic approach in anti-cancer therapy. In our review we provide evidence for both tumor supporting and tumor inhibiting functions of scavenger receptors expressed on TAMs. We focused on the key differences in the prognostic and functional roles of SRs that are specific for cancer types. We highlighted perspectives for inhibition of tumor-promoting SRs in anti-cancer therapy.

## 1 Introduction

Tumor-associated macrophages (TAMs) are key innate immune cells that control intratumoral inflammation, cancer cell proliferation, migration and metabolism, angiogenesis, and extracellular matrix composition (1–6). Major sources of TAMs are resident tissue macrophages as well as monocyte-derived macrophages, intensively recruited into the growing tumor by chemotactic factors, like CCL2 (4, 7, 8) The are two major vectors of macrophage polarization: M1-type, classically activated, pro-inflammatory, and M2-type, alternatively activated, generally considered as anti-inflammatory or tolerogenic macrophages (4–6). The classification based on the M1/M2 dichotomy is traditionally used as simplified schema to distinguish between two major directions of macrophage activity. M1 macrophages play an important role in the innate immune response, while M2 macrophages are involved in tissue repair, as well as in the progression of many types of cancer (9, 10).

Within tumor tissues, TAMs interact with cancer cells and with other cell types in tumor microenvironment (TME) not only by secreting different cytokines, chemokines and growth factors, but also by clearance of dying cells, soluble mediators and matrix components mediated by scavenger receptors (SRs). SRs can recognize and internalize high range of unwanted-self ligands including cytokines, growth factors, modified lipoproteins, apoptotic cells, as well as non-self ligands including bacteria, viruses and fungi (11–13).

SRs are large superfamily of transmembrane proteins with high structural diversity (13–15). SRs are categorized into classes A-L depending on their structure, cell-type specific expression and recognition of host-derived ligands (13, 16, 17). Functional diversity of SRs are crucial for numerous biological processes such as endocytosis, phagocytosis, cell adhesion, nutrient exchange and waste clearance, as well as immunity processes, e.g. inflammation regulation and antigen presentation (13, 14).

In tumors, SRs can be expressed by both tumor cells (TCs) and by components of the TME including macrophages, monocytes, endothelial cells and dendritic cells (15, 18, 19). Most commonly used SRs for the identification of TAM subpopulation in different types of tumors include CD68, CD163, CD204 and CD206 (8). Both tumoricidal M1 and tumor-promoting M2 macrophages express CD68, while M2 polarization can be identified by CD163, CD204 and CD206 biomarkers (8). However, this nomenclature does not fully reflect all phenotypic diversity of TAMs that can combine M1 and M2 features and functions (3).

In this review, we focus on functional activity of scavenger receptors expressed by TAMs in tumor. We summarize the latest knowledge on the functional activity of TAM-expressing scavenger receptors in the TME. We discuss how expression of scavenger receptors in TAMs can be used for the evaluation of prognostic value in numerous cancers.

## 2 Scavenger receptors expressed by TAMs

**Figure 1:**
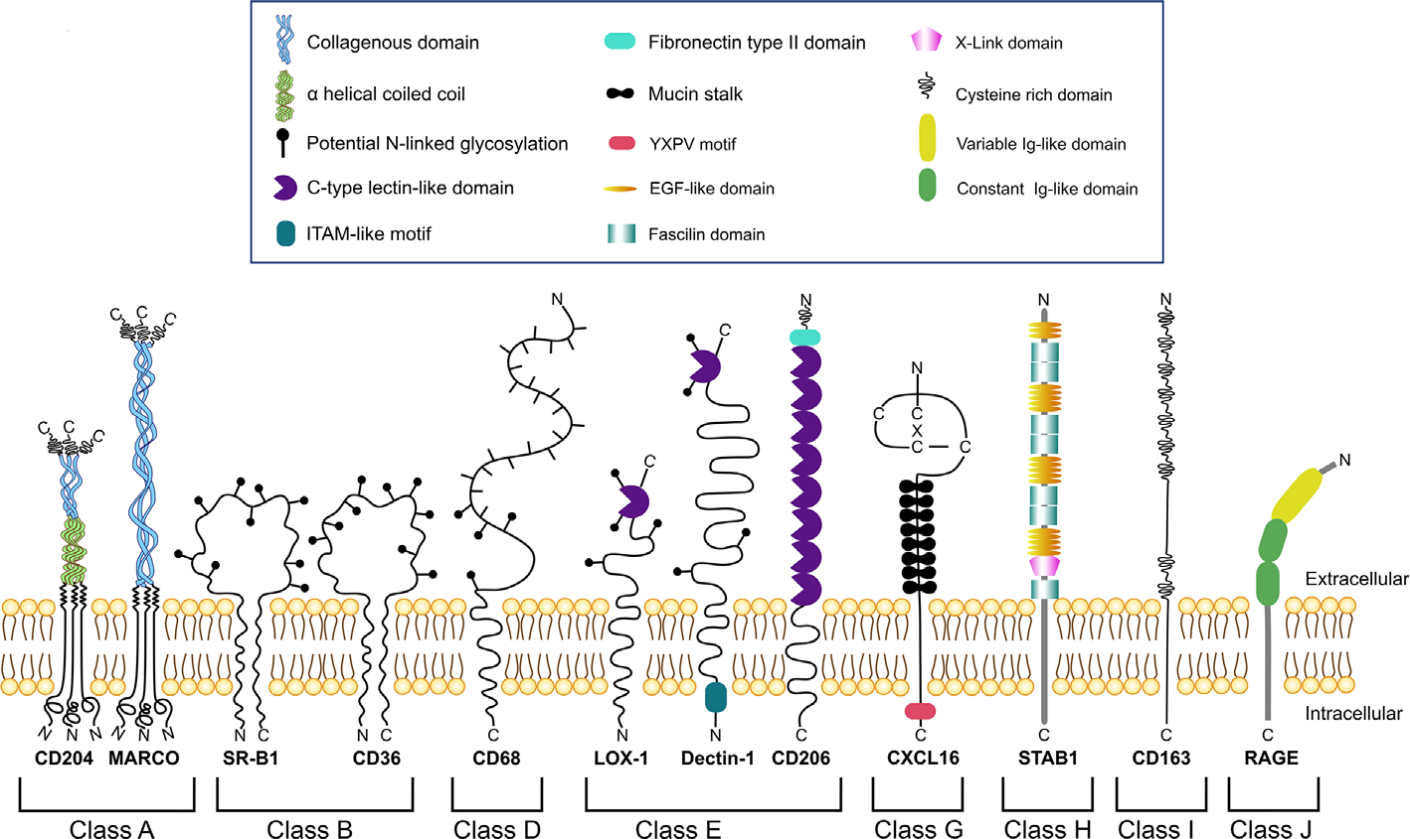
Schematic representation of different classes of scavenger receptors expressed by TAMs.

Several SRs play essential role in the regulation of TME where they can be expressed by TAMs, NK cells, dendritic cells, neutrophils, B cells, endothelial cells, epithelial cells and cancer cells (13). TAM-expressing scavenger receptors are structurally heterogeneous proteins that consist out of diverse structural domains including collagenouse domain, C-type lectin-like domain, fibronectin domain, EGF-like and others (**Figure 1**). SRs expressed by TAMs are involved in diverse signaling pathway and have a predictive value for tumor progression (**Table 1**) (23, 25–27, 29, 30, 48).

**Table 1:** The function of TAM-expressing scavenger receptors in the TME.

Scavenging receptors	Ligands	Function/mechanism	Correlation with clinical parameters
Class A			
SR-A1 (CD204)	Lipopolysaccharide (LPS), lipoteichoic acid (LTA) and bacterial CpG DNA	Anti-tumor: Suppression of tumor growth and angiogenesis *via* inhibition of COX-2, SDF1, VEGF, and MMP9 expression and down-regulation of JNK/ERK/IκB/NFκB signaling pathway in LLC tumors, as well as inhibition of macrophage polarization (20) and monocyte recruitment (21, 22).	Correlation with better RFS in prostate cancer (23). Negative correlation with human lung cancer progression (21). Positive correlation with short OS and RFS in colorectal cancer (24). Correlation with better survival rate and less recurrence in glioma (25).
		Pro-tumor: Promotion of proliferation, migration and invasion of MCF7, T47D, SKBR3, MDA-MB-231, ID8 cell lines *in vitro* (26, 27). Induction of lung metastasis in a mouse model of pancreatic adenocarcinoma (26).	
SR-A6 (MARCO)	Oxidized lipids, unopsonized particles, bacteria, integrins	Anti-tumor: Clearance of colon carcinoma cells *via* the SYK-PI3K-Rac1 signaling pathway (28).	Positive correlation of the number of MARCO+ TAMs with DFS and OS in pancreatic cancer and squamous cell carcinoma (29, 30). Increase amount of MARCO+TAMs associates with prolonged OS in human HCC (31).
		Pro-tumor: Association with high expression of tumor-supporting genes in NSCLC and glioblastoma (30, 32). Activates immunosuppressive phenotype of TAMs (33).	
Class B			
SR-B3 (CD36)	Thrombospondin-1, long-chain free fatty acids, ox-LDL, advanced glycation endproducts (AGE), collagens I and IV	Anti-tumor: N/A	N/A
		Pro-tumor: Promotion of tumor growth *via* up-regulation of pro-tumor genes, M2-signature genes in TAMs and enhancing TAM infiltration in lymphoma (34). Supporting tumor development through activation of S100A4-PPAR-γ pathway in TAMs in breast cancer and fibrosarcoma (35). Increasing of tumor growth *via* promotion of TAM infiltration in tumor in breast cancer (36).	
Class D			
SR-D1 (CD68)	oxLDL, phosphatidylserine, apoptotic cells, malaria sporozoite	Anti-tumor: N/A	Correlation with to worse prognosis in glioblastoma, kidney renal clear cell carcinoma, lower-grade glioma, hepatocellular carcinoma, lung squamous cell carcinoma, thyroid carcinoma, thymoma and a favorable prognosis in chromophobe renal cell carcinoma, LSCC, breast cancer (37, 38). Correlation with recurrence in cutaneous melanoma (39). Positive correlation with favorable neoadjuvant chemotherapy responses in osteosarcoma (40). Correlation with anti-tumor TAM phenotype in melanoma (41).
		Pro-tumor: promotion of angiogenesis in LSCC (37).	
Class E			
SR-E1 (LOX-1)	Ox-LDL, apoptotic cells, gram-positive and gram-negative bacteria, acute phase C-reactive proteins, HSP	Anti-tumor: N/A	Decreased amount of LOX-1+ TAMs is associated with poor OS in colorectal cancer (42).
		Pro-tumor: Promotion of M2 TAM polarization *via* PI3K/Akt/mTOR signaling in HNSC (43).	
SR-E2 (Dectin-1)	β-1,3-glucan, galectin-9, annexins, vimentin, N-glycan	Anti-tumor: Supporting of TAM tumoricidal activity against lymphoma and ovarian adenocarcinoma (44).	Correlation with shorter patient recurrence free survival and overall survival in cell renal cell carcinoma (45).
		Pro-tumor: Promoting pancreatic cancer progression *via* increasing TAM infiltration, reprogramming TAMs toward M2 phenotype and reduced T-cell infiltration (46).	
SR-E3 (CD206)	Collagens, N-acetylgalactosamine (GalNAc)	Anti-tumor: Suppression melanoma growth *via* activation of tumoricidal T cells (47).	Positive correlation with tumor relapse and metastasis after chemotherapy in breast cancer (48) and correlation with worse clinical prognosis in OSCC (49). Increased amount of CD206+TAMs associated with improved overall survival in cutaneous melanoma (47).
		Pro-tumor: Promoting proliferation and invasion of OSCC cells by producing EGF (49).	
Class G			
SR-G1 (CXCL16)	oxLDL	Anti-tumor: overexpression in colorectal cancer cells causes TNFα-mediated apoptosis (50).	Associated with aggressive pathologic phenotypes, the higher TNM staging and lymph node metastasis in papillary thyroid cancer (51). Decrease of the overall survival due to *CXCR6* overexpression, receptor of CXCL16 (52).
		Pro-tumor: enhancing tumor cell migration, invasion, proliferation and promoting M2 TAM polarization (51–54).	
Class H			
SR-H1 (STAB1)	ac-LDL, placental lactogen, SPARC, advanced glycation end products, apoptotic cells, microparticles from gram-positive and negative bacteria	Anti-tumor: N/A	Positive correlation with long DSS and favorable prognosis in early stage I CRC patients (55). Correlation with poor OS, RFS, tumor stage and histological grade in urothelial carcinoma of the bladder and rectal cancer (55, 56).
		Pro-tumor: Supporting breast cancer progression through activation of PKCβ expression in TAMs resulted in SPARC uptake from TME by TAMs (57).	
Class I			
SR-I (CD163)	Haptoglobin-hemoglobin complex	Anti-tumor: N/A	Correlation with tumor grade in breast cancer (58). Positive correlation with severe prognosis of myeloma, gastroesophageal adenocarcinoma, triple negative breast cancer (59–61). Correlation with lymph node metastasis poor prognosis in breast cancer (62). Negative correlation with recurrence and poor overall survival in primary melanoma (39).
		Pro-tumor: Promoting TAM polarization toward tumor-supporting TAM phenotype in cholangiocarcinoma (63). Induction of tumor progression *via* production of IL-6 and CXCL2 and activation of STAT3 in fibrosarcoma (64).	
Class J			
RAGE	AGEs, HMGB1, S100 proteins, amyloid-beta peptide, dsDNA and dsRNA	Anti-tumor: AGEs and HMGB1 promote M1 polarization in macrophages and TAMs, respectively (65)^,93,94^. HMGB1 exposure to M1 macrophages abrogates invasion of gastric tumor cells and growth of endothelial cells (66).	HMGB1 and CD163 positive macrophages were found as detrimental prognostic factors for OS in laryngeal squamous cell carcinoma (67). High KRAS^G12D^ expression in CD68^+^ cells in PDAC patients correlated with worse OS rates (68).
		Pro-tumor: HMGB1 activation of RAGE in M2 macrophages promotes invasion of gastric tumor cells (66), production of VEGF (66, 67, 69) and angiogenesis (66, 67). Mediates KRAS^G12D^ uptake, which promotes M2 polarization of TAMs (68).	

### 2.1 Class A scavenger receptors

Scavenger receptors of class A (SR-A) are transmembrane proteins containing a collagen-like domain with collagen-binding activity (15). SR-A family comprises five members: SR-A1 (CD204*)*, SR-A3, SR-A4, SR-A5, and SR-A6 (MARCO), which recognize a variety of ligands such as LPS, LTA and integrins. SR-A are implicated in several pathologies including atherosclerosis, infectious diseases and cancer (13, 70, 71). In most of studies the question about tumor-specific ligand of CD204 was not addressed experimentally. The expression of SR-A was found on monocytes, macrophages, dendritic cells (DCs), mast cells and endothelial cells (71, 72). Out of five SR-A family members, only CD204 and MARCO have been found to be expressed by TAMs (21, 22, 25).

#### 2.1.1 SR-A1/CD204

SR-A1 (also known as CD204, or MSR1) is a pattern recognition receptor expressed primarily on macrophages, and is involved in the inflammatory responses and tumorigenesis (21, 25, 73). CD204 has a dual role in cancer progression (21, 22, 26, 27, 74).

**Figure 2:**
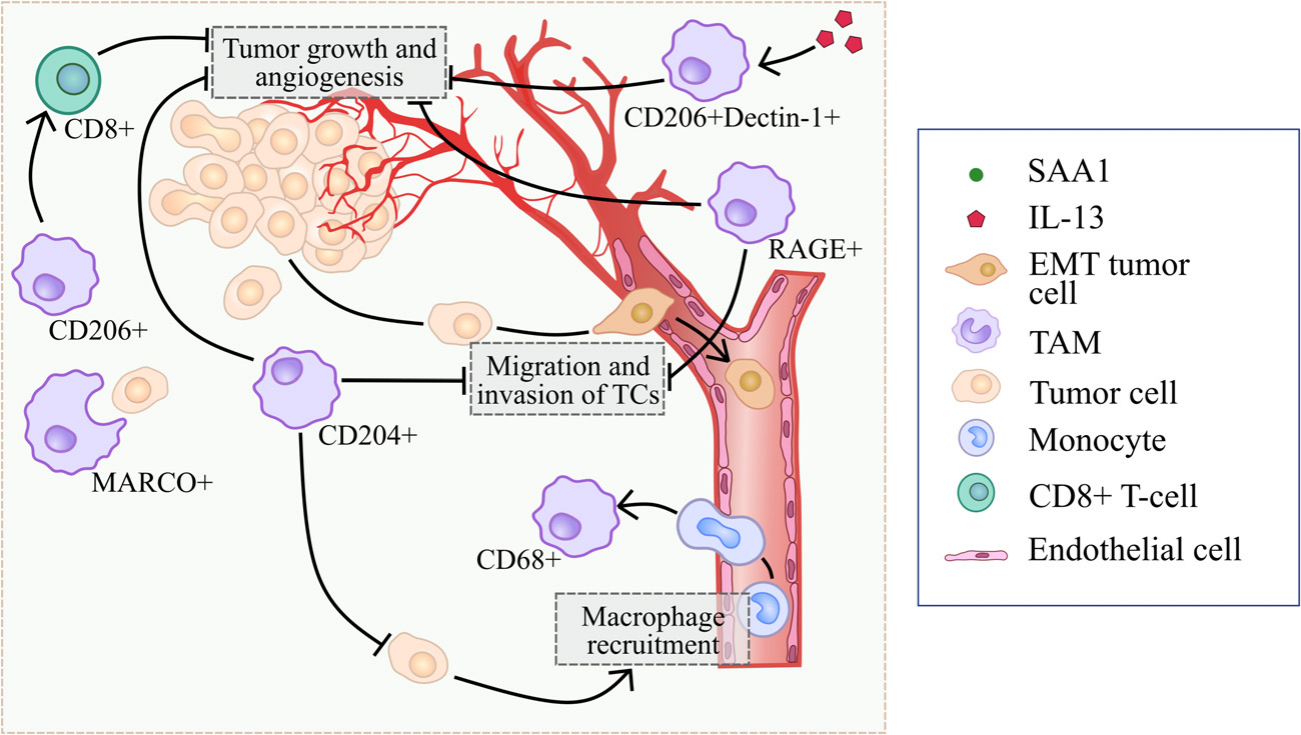
Summary of processes that are regulated by scavenger receptors in tumor-suppressing microenvironment.

The mechanisms of CD204 anti-tumor activity in TME include inhibition of macrophage infiltration, inhibition of tumor cell migration and invasion, as well as suppression of tumor angiogenesis (21, 22) (**Figure 2**). In a mouse model of LLC, bone marrow-derived cells transplanted from CD204 KO (knock-out) mice into WT mice enhanced tumor growth and angiogenesis through elevated COX-2, SDF1, VEGF and MMP9 expression in tumor (21). CD204 deficiency activated recruitment of CD68+ and F4/80+ macrophages into tumor mass by upregulation of MCP-1 in the CD204 KO bone marrow transplantation model (22). Peritoneal macrophages isolated out of CD204 KO mice significantly enhanced the migration and invasion of lung cancer cells *in vitro* (22). Moreover, CD204 suppresses tumor development through the upregulation of serum amyloid A1 (SAA1) expression in TAMs *via* JNK/ERK/IκB/NFκB signaling pathways (22). CD204 deficiency promoted tumor growth, angiogenesis and TAM infiltration *via* skewing TAM phenotype toward M2 in murine glioma model (25). Tumor volume as well as the expression of angiogenic factors CD31, CD34, IB4 and VEGF were significantly elevated in CD204−/− mice in comparison with CD204+/+ mice in glioma model (25). In CD204−/− glioma, the number of VCAM1+ TAMs and CCR2+ TAM precursor cells was significantly elevated compared to CD204+/+ glioma (25). *In vitro*, CD204 deficiency resulted in the increased expression of M2-like markers (*MMP2, TGFβ, MRC2, MGL1, FIZZ1*), but no M1-like marker (TNFα) in the presence of GL261 glioma cells (25). Meta-analysis of patients with prostate cancer showed that increased expression of CD204 significantly correlated with better recurrence-free survival (RFS) (23). Immunohistochemical (IHC) analysis demonstrated that high expression of CD204 correlated with better survival rate and less recurrence than those with less CD204 expression in patients with glioma (25).

**Figure 3:**
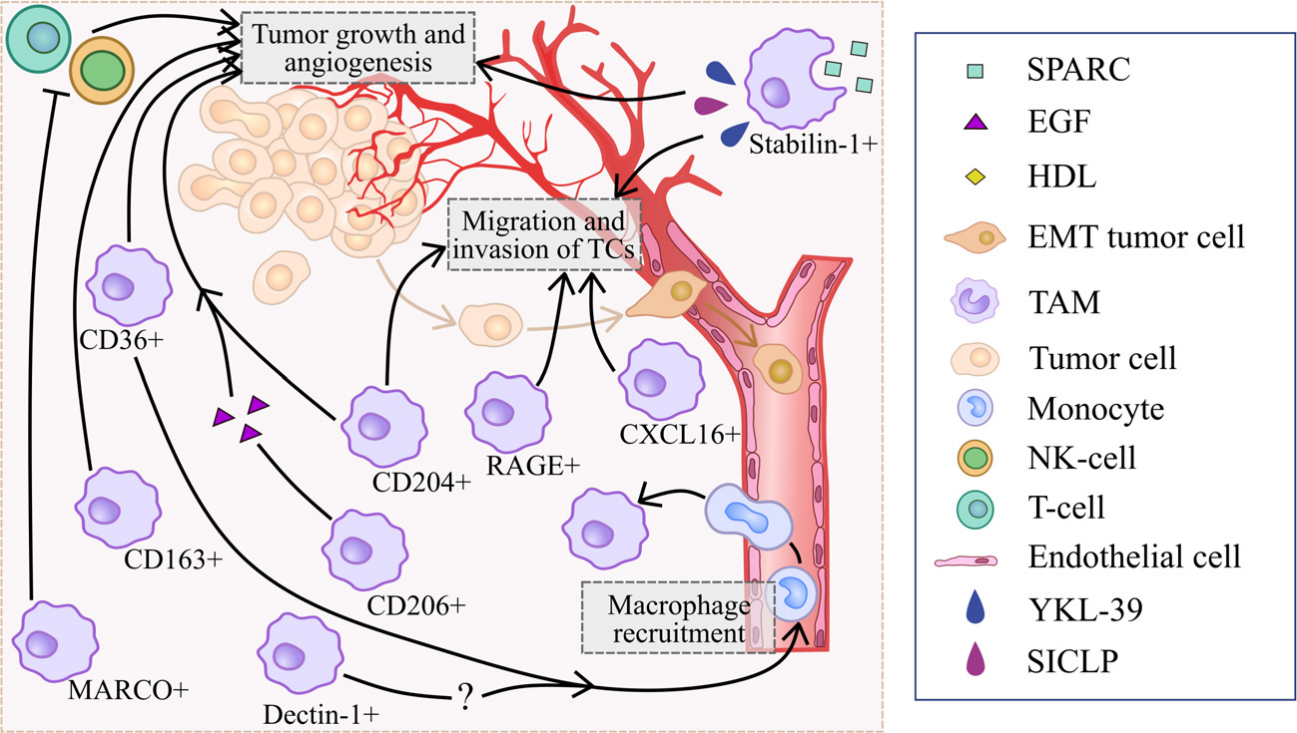
Summary of processes that are regulated by scavenger receptors in tumor-supporting microenvironment.

However, controversial data also exist (**Figure 3**). Several studies demonstrated that CD204+TAMs promote tumor development and correlate with worse prognoses in prostate cancer, lung cancer, colorectal cancer, cervical cancer, breast cancers and oral squamous cell carcinoma patients (24, 26, 27, 74–76). Tumor-supporting function of CD204+ TAMs was demonstrated for lung cancer and glioma, however anti-tumor function was shown for breast cancer, ovarian cancer and pancreatic cancer (21, 24, 25, 27, 74–76). CIBERSORT analysis of CD204 mRNA expression obtained from TCGA database demonstrated that high CD204 expression correlated to high proportions of M2 macrophages and the expression of immunosuppressive molecules, including HIF1A, FAP, IL-10, and TGFB1 in breast cancer (27). CD204 KO macrophages reduced tumor cell invasion *via* TLR-dependent pathways upon co-culture with ID8 (ovarian cancer cell line) and Panc02 (pancreatic adenocarcinoma cell line) (26). Macrophage-specific loss of CD204 significantly reduced lung metastasis in a mouse model of pancreatic adenocarcinoma (26). *In vitro* CD204+ TAMs promoted proliferation, migration and invasion of MCF7, T47D, SKBR3 and MDA-MB-231 breast cancer cell lines (27). High CD204 expression in TAMs correlates with short overall survival (OS), disease-free survival (DFS) and RFS in colorectal cancer, cervical cancer, breast cancers and oral squamous cell carcinoma (OSCC) (24, 74–76). CD204 expression is associated with T stage, nodal involvement, lymphovascular invasion and tumor relapse after surgery in lung adenocarcinoma (25, 77). In prostate cancer, high CD204 protein expression in the main tumor area predicted a worse prognosis, while CD204 expression in seminal vesicle invasion area was positively associated with the biochemical recurrence (78).

In summary, majority of reports show that CD204 correlates with good prognosis in prostate cancer and glioma, and with worse prognosis in colorectal cancer, cervical cancer, breast cancers, oral squamous cell carcinoma, lung cancer and prostate cancers. Murine experimental systems demonstrated both tumor-promoting and tumor-inhibiting role of CD204+ TAMs (**Figures 2**, **3**).

#### 2.1.2 SR-A1/CD204

SR-A6 (also known as macrophage receptor with collagenous structure, MARCO) is another member of the SR-A family that is also expressed by macrophages and is involved in clearance of cancer cells, in regulation of epithelial-mesenchymal transition (EMT), in the interferon-alpha response, and in antigen presentation (28, 79).

**Figure 4:**
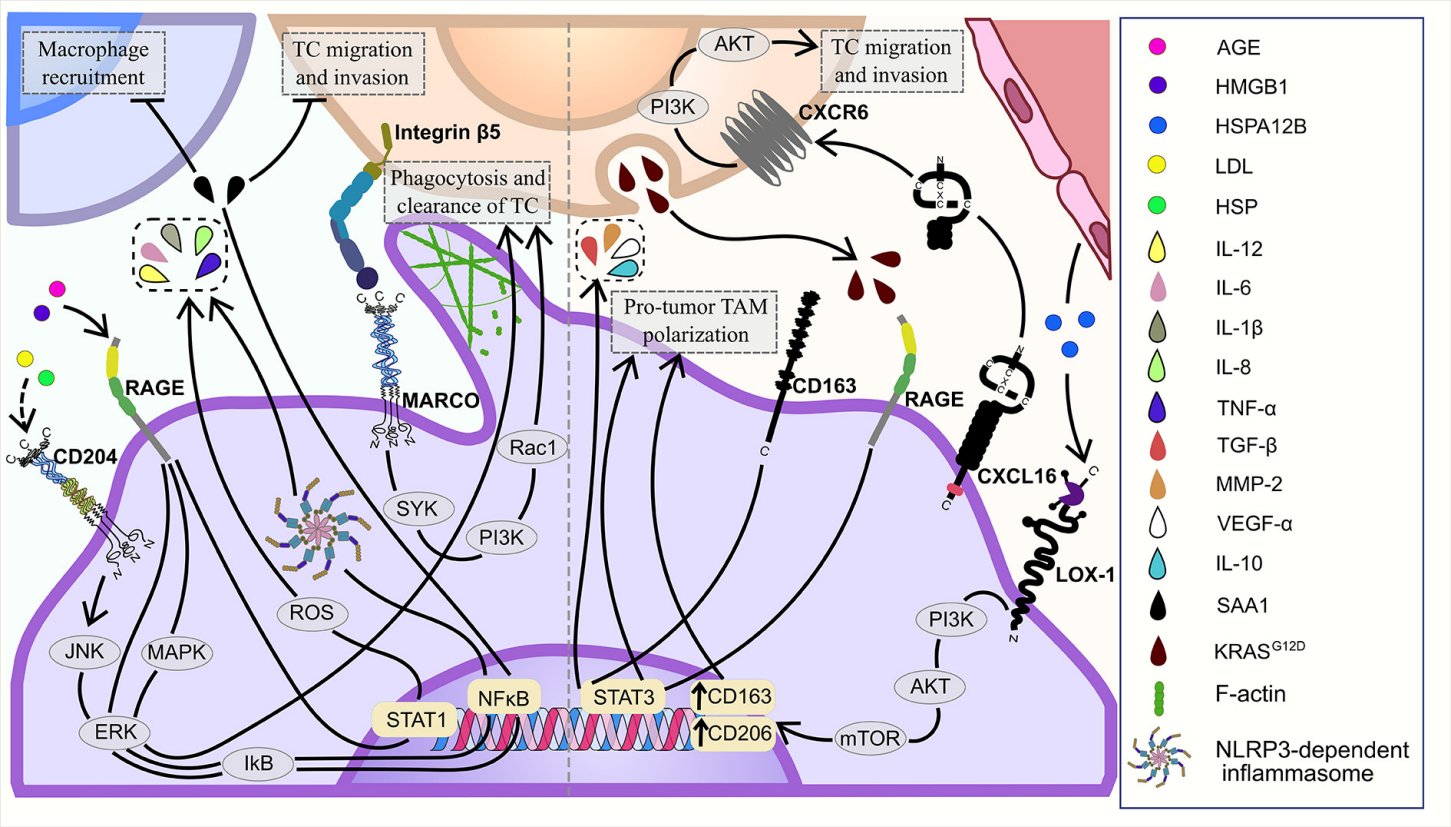
Major signaling pathways of TAM-expressing scavenger receptors in the TME: tumor-supporting and tumor-inhibiting.

*In vitro*, TAMs suppress tumor development utilizing MARCO to phagocytose cancer cells (**Figure 2**) (28). MARCO overexpression in peritoneal murine macrophages led to the increased expression of SYK, PI3K and Rac-1, and facilitated macrophage-mediated phagocytosis of SL4 (colon carcinoma cell line) *via* binding to integrin β5 on cancer cells and activation of SYK-PI3K-Rac1 signaling pathway in TAMs in the co-culture system (28) (**Figure 4**).

Several studies on clinical material revealed tumor-supporting phenotype of MARCO-expressing TAMs (30, 32, 33). Single-cell transcriptomic analysis of glioblastoma demonstrated that a cluster of MARCO+ TAMs coincides with high expression of genes involved in epithelial-mesenchymal transition, angiogenesis, glycolysis, hypoxia and low expression of genes associated with interferon-alpha response, interferon-gamma response, allograft rejection, and TNFα signaling (32). MARCO+ TAMs support tumorigenesis by activating immunosuppression in the TME. Transcriptomic analysis of non-small cell lung cancer (NSCLC) samples showed that MARCO expression significantly correlated with gene expression of immunosuppressive TAM-related genes (CD163, MSR1, IL4R, CHIA, TGFB1, and IL10), genes involved in T-cell regulation (FOXP3, TGFB1, IL10, EBI3, PDCD1, and CTLA4) and genes encoding immune checkpoint molecules (PD-L1, PD-L1, VISTA, PD-1, and CTLA4) (30). High infiltration of MARCO+ TAMs in tumor was associated with worse OS and DFS in patients with squamous cell carcinoma (30) and pancreatic cancer (29).

MARCO-expressing TAMs suppressed tumoricidal activity of T cells and NK cells through skewing TAM phenotype toward anti-inflammatory one (**Figure 3**). *In vitro* MARCO-expressing TAMs suppressed activation, proliferation and IFNγ production in T cells, resulted in inhibition of T-cell killing activity towards NSCLC tumor cells (33). Moreover, human PBMC-derived MARCO+ TAMs inhibited migration, degranulation, proliferation and IFNγ production in NK cells (33). MARCO+ macrophages cultured with lung cancer cell lines displayed decreased expression of pro-inflammatory cytokines (TNFa, IL1B and IL12B) and increased expression of anti-inflammatory molecules (IL10, MRC1, COX2, TIMP1, and FIZZ1) (33). High expression of MARCO in TAMs from the tumor tissues was associated with increased OS in patients with hepatocellular carcinoma (HCC) (31).

Thus, role of SR-A family members depends on the tumor context. Despite strong tumor-supporting activity identified for several members of SR-A family expressed by TAMs (26, 27, 30, 32, 33), solid body of evidence is available for SR-A1 and SR-A6 demonstrating their anti-tumor action that depends primarily on the cancer types (20–22, 28) (**Figure 3**, **Table 1**).

### 2.2 B scavenger receptors

Class B scavenger receptors includes the following members: SR-B1, SR-B2 and SR-B3. Structurally Class B scavenger receptors are constructed out of two transmembrane domains flanking an extracellular loop, with both the N- and C-termini located within the cytoplasm (80). Class B scavenger receptors mediate transport of cholesterol and lipids, and are involved in tumor development (34, 35, 81). The role of TAMs expressing SR-B1 and SR-B3 was demonstrated in several types of cancers, including liposarcoma, nasopharyngeal carcinoma, breast cancer, colon cancer and prostate cancer (35, 36, 82).

#### 2.2.1 SR-B1

Scavenger Receptor Class B Type 1 (SR-B1) is a transmembrane protein that act as a major high-density lipoprotein (HDL) receptor (81, 82). SR-B1 is expressed by endothelial cells, smooth muscle cells, keratinocytes, adipocytes, tumor cells and macrophages (19). In the TME, SR-B1 participates in HDL metabolism and promotes invasion, proliferation and metastasis of tumor cells (82). In macrophages, SR-B1 regulates cholesterol metabolism through selective uptake of HDL-cholesterol and cholesteryl esters (83). In a syngeneic mouse model of prostate cancer, knock out of SR-B1 inhibited HDL-mediated tumor growth and progression (84). In SR-B1−/− mice had lower levels of total cholesterol and HDL-cholesterol. SR-B1−/− mice developed smaller tumor compared to SR-B1+/+ mice, and SR-B1−/− mice showed also the decreased survival (84). Application of HDL-mimetic nanoparticles that interacted with SR-B1 reduced tumor growth in a mouse xenograft model for human nasopharyngeal carcinoma (82). Thus, the selective uptake of HDL-cholesterol by SR-B1 in macrophages is a promising pathway for pharmacological inhibition of pro-tumor TAM actions. SR-B1 activity in macrophages is mediated by Src/PI3K/Akt/Rac1 and PPARγ/LXRα signaling pathways (85, 86). The data about the role of TAM-expressing SR-B1 in cancer are limited, but SR-B1 expression was found in head and neck cancer, lung cancer, prostate cancer and breast cancer, where it positively correlates with the tumor aggressiveness and poor prognosis (82, 87, 88).

#### 2.2.2 SR-B3/CD36

SR-B3 (also known as CD36) is expressed on monocytes, macrophages, platelets, endothelial cells, adipocytes (89, 90). CD36 mediates lipid uptake, ligand, clearance of apoptotic cells and cell-cell adhesion (90–92). CD36-expressing macrophages facilitate tumor progression, pro-tumor TAM polarization and mediate fatty acid uptake from TME (**Figure 3**) (35, 36, 93).

CD36 regulates polarization of TAMs towards pro-tumor phenotype and promotes tumor growth *via* regulation of fatty acid (FA) metabolism (94, 95). CD36 was demonstrated as a major SR on macrophages involved in the lipid uptake and accumulation, FA oxidation and oxidative phosphorylation (94, 95). In TME extracellular free fatty acids, including palmitic acid, oxLDL or oleic acid, are transported into cells *via* membrane-associated CD36 and promoted tumor growth and metastasis (94–97). Essential feature of CD36 is that its endocytic function is linked to the inflammatory pathways. In macrophages CD36 is involved in diverse signaling cascade including NF-κB pathway, TLR1/2 signaling, TLR4 signaling and NOD-, LRR-, and pyrin domain-containing protein inflammasome pathways (98).

In co-culture of human PBMCs and tumor cells CD36 regulates macrophage response by enhanced lipid uptake and increased expression of pro-tumor genes in modeled TAMs (Arg1, Ccl2) (34). Subcutaneously injection of CD36-KO TAMs in a mouse model of lymphoma decreased tumor volume, impaired TAMs infiltration into tumor site, increased expression of M1-signature genes and decreased expression of M2-signature genes (34). CD36 in TAMs mediates FA uptake through S100A4-PPAR-γ axis that promotes tumor growth in a mouse models of breast cancer and fibrosarcoma (35). In the mouse model of breast cancer CD36 regulates TME *via* clearance of tumor-derived miR-375, a prominent tumor suppressor (36, 93). In co-culture system of MCF-7 cells and human PBMCs, apoptotic tumor cell-derived miR-375 binds to LDL and is scavenged by TAMs *via* CD36 receptor resulting in increased macrophage migration and infiltration into tumor (36).

Association of CD36 with worse prognosis was demonstrated in several human cancers including bladder cancer, glioblastoma, oral carcinoma and gastric cancer (97, 99). However, currently prognostic significance of CD36 expressed specifically on TAMs is still unclear.

### 2.3 Class D scavenger receptors

Scavenger receptor SR-D1 (also known as CD68) is the only known class D scavenger receptor that is highly specifically expressed on macrophages and other mononuclear phagocytes but not on other cell types, even of myeloid origin. CD68 is the major biomarker for the quantification of total TAM amounts. CD68 is also a well-established pan-macrophage marker used as a cancer-associated diagnostic and prognostic marker (8, 13).

CD68+TAM infiltration and accumulation in tumor results in tumor progression and adverse prognosis in numerous cancers (8, 37, 38). In our recent review we have summarized data from large number of studies on patients with 5 types of cancer: breast, colorectal, lung, ovarian and prostate (9). Number of studies on patients representing diverse genetics, life style and geographical localizations indicate that amount of intramural CD68+TAMs positively correlates with negative prognosis, distant hematogenous and local lymphatic metastasis in breast, lung, ovarian and prostate cancers. However, amount of intramural CD68+TAMs showed negative correlation with the bad outcome in patients with colorectal cancer. Recent analysis of Genotype-Tissue Expression datasets (TCGA) and immunohistology have demonstrated that high expression of CD68 was correlated with worse prognosis in glioblastoma, renal clear cell carcinoma, lower-grade glioma, HCC, lung squamous cell carcinoma, thyroid carcinoma, and thymoma, but with favorable prognosis in chromophobe renal cell carcinoma (38). In laryngeal squamous cell carcinoma (LSCC), CD68+ cells were involved in angiogenesis and correlated with worse prognosis (37). High expression of CD68 was associated with CD34+ cells in tumor and low 5-year DFS in 45 patients with LSCC from China (37). High amounts of CD68+ TAMs in tumor nest correlated with recurrence in 184 cutaneous melanoma patients from Finland (39). Number of CD68+ TAMs in tumor stroma were positively correlated with tumor size in breast cancer both in 144 patients from Sweden and in 60 patients from Egypt (100, 101). Controversially, in melanoma, CD68+ TAMs characterized by M1 phenotype, however, not statistically significant correlations were found for the total amount of CD68+TAMs and clinical parameters of melanoma progression in patients (41). RNA-seq and IHC analysis of 57 human melanoma samples showed that CD68+ TAMs were associated with increased iNOS and arginase expression (41). In human osteosarcoma, elevated expression levels of macrophage and CD4 T-cell markers (defined as CD4/IFNGR2/CD68/CSF1R signature) was associated with favorable neoadjuvant chemotherapy responses (40).

In several independent studies amount of intratumoral CD68+ TAMs were indicative for reduced tumor growth and better prognosis (8). CD68+ macrophage infiltrates correlated with better RFS in 468 patients with ER-negative tumors from Scotland (102). In a Norway study of 553 primary NSCLCs CD68+ expression correlated with favorable NSCLC-specific survival (103). Correlation of high expression of CD68 with favorable prognosis was also demonstrated in colorectal cancer (104, 105). Thus, total amount of CD68+ TAMs is a potential prognostic biomarker that can predict negative scenario in progression for majority of human cancer types, however opposite correlations were identified for specific cancer types, in particular colorectal cancer, raising urgent question about intrinsic anti-tumor activates of TAMs that cannot be converted by growing tumor. However, functional role of CD68 in inflammation and carcinogenesis is not sufficiently understood despite its routine application as an immunochemical marker of TAMs.

### 2.4 Class E scavenger receptors

The class E of scavenger receptors comprises SR-E1, SR-E2, SR-E3 and SR-E4 members (13). The class E SRs belong to a subfamily of NK cell C-type lectin-like (CLEC) receptor family that plays role in diverse biological processes such as immune response, antigen presentation and phagocytosis (13). Several members of the Class E SR family are expressed in TAMs and involved in tumor progression (42, 43).

#### 2.4.1 SR-E1/LOX-1

SR-E1 (also known as LOX-1) is mainly expressed by endothelial cells, but is also found on smooth muscle cells, cardiomiocytes, adipocytes, platelets and on TAMs (106). LOX-1 participates in multiple physiological and pathological processes, including lipid metabolism, cholesterol biosynthesis and tumorigenesis (107, 108). In tumors, LOX-1 regulates macrophage polarization (43). Correlation analysis of TCGA data, single-cell RNA-seq data and *in vitro* models showed, that TAMs increased the uptake of heat shock protein HSPA12B by LOX-1 that resulted in the activation of PI3K/Akt/mTOR signaling and enhanced M2-type marker expression (CD163 and CD206) in TAMs in head and neck squamous cell carcinoma (HNSC) (43) (**Figure 4**). At the moment, the detailed mechanism of LOX-1+ TAM activity in the TME is not well defined, but the prognostic value of LOX-1+ TAMs was found in colorectal cancer (42). IHC analysis demonstrated that low expression on TAMs was associated with poor OS in patients with colorectal cancer (42).

#### 2.4.2 SR-E2/Dectin-1

SR-E2 (also known as Dectin-1) is a C-type lectin receptor that is involved in large number of biological processes such as phagocytosis, activation of signaling pathways, generation of reactive oxygen species (ROS) and production of cytokines (109). Dectin-1 is encoded by Clec7a gene and primary expressed on the surface of the myeloid-monocytic lineage cells including macrophages, but can be also found on neutrophils, dendritic cells, and on a minor subpopulation of splenic T cells (46, 110). Dectin-1 is an innate immune receptor playing role in anti-fungal immune response. In cancer, dectin-1 regulates immune microenvironment and has an ambiguous function in tumor progression (44, 46, 111).

IL-13-activated macrophages expressing both dectin-1 and mannose receptor (MR) inhibited T-cell lymphoma and ovarian adenocarcinoma progression *via* binding to tumoral sialic acid (44). Dectin-1 and MR interaction of with sialic acid enhanced antitumor effect of IL-13- activated macrophages *in vitro* (44). Depletion of dectin-1 and MR in IL-13-activated macrophages resulted in inhibition of TAM tumoricidal activity and decrease in death of Jurkat (human T-cell leukemia cell line) and EL4 (murine T-lymphoma cell line) tumor cells (44).

Dectin-1 promotes tumor progression *via* the regulation of immune microenvironment of human OSCC (111). Dectin-1 deficiency decreased the amount of IL-1β+ cells, Tregs, MDSC cells and PD-1 induction in CD8+ T cells resulted in slower dysplasia progression and lower number and size of tumors in mouse model of OSCC (111). Dectin-1 promotes pancreatic ductal adenocarcinoma (PDA) progression by enhanced TAM infiltration and by reprogramming TAMs towards M2 phenotype (46). In a mouse model of PDA, Clec7a deletion significantly reduced the infiltration of PDA with F4/80+, CD206+ and Arg1+ TAMs, as well as upregulated MHCII, TNF-α and iNOS expression in tumor (46). Moreover, depletion of Clec7a in macrophages *in vivo* elevated infiltration by CD4+ and CD8+ T cells selectively in wt hosts, but not in Clec7a−/− hosts, indicating that dectin-1-expressing macrophages drive T cell suppression in PDA (46). In renal cell carcinoma, high expression of tumor cell-derived but not TAM-derived dectin-1 was associated with shorter RFS and OS (45).

#### 2.4.3 SR-E3/CD206

Scavenger receptor SR-E3 (also known as CD206) is a C-type lectin that mediates antigen presentation, endocytosis, phagocytosis and immune homeostasis (112, 113). It is commonly accepted that CD206 is a marker of tumor-supporting M2 phenotype of TAMs, but recent studies demonstrated controversial activity of CD206+ TAMs in tumor (47–49).

CD206+ TAMs produced EGF to promote OSCC progression *in vitro* (49). Proliferation and invasion of OSCC cells cultured with conditioned medium of CD206+ TAMs were strongly enhanced by EGF (49). CD206 mediated breast cancer post-chemotherapy progression (48). In mouse model of breast cancer high expression of CD206+F4/80+ TAMs was associated with tumor relapse and lymph node metastasis after cyclophosphamide treatment (48). Number of CD206+ TAMs positively correlated with worse clinical prognosis in OSCC, CRC, lung cancer (49, 114, 115).

In contrast, CD206+TAMs were shown to program T cells to attack melanoma tumor cells (47). Antigen cross-presentation in tumor remains to be a challenging issue for development of anti-cancer therapy. Primary human as well as mouse CD206+ macrophages were recently shown to be efficient in functional cross-presentation of soluble self-Ag and non-self-Ag, including tumor-associated Ag (TAA) (47). CD11b+CD206+ TAM were found to express a unique cell surface repertoire, promoting antigen cross-presentation and antigen-specific activation of CD8+ T cells. In murine tumor models, the levels of cross-presenting CD206+ TAMs correlated with reduced tumor burden (47). CD206+ TAMs also correlated with improved overall survival of cutaneous melanoma patients. It is an intriguing question to be addressed in future, which self-antigens can be presented to the adaptive immunity in different types of solid cancers by CD206 TAMs, and what is the impact of this process in overall role of CD206 in cancer (47).

### 2.5 Class G scavenger receptors

#### 2.5.1 SR-G/CXCL16

CXCL16 (also known as SR-G1, or SR-PSOX) is a scavenger receptor mediating endocytosis of oxidized low-density lipoproteins (OxLDL). CXCL16 is primarily expressed on macrophages and dendritic cells. CXCL16 exists in both transmembrane and soluble forms. The soluble form acts as a chemokine specifically binding to CXCR6, and the transmembrane SR-G1 represents an adhesion molecule for CXCR6-expressing cells (116, 117).

CXCL16 has been shown to have a pro-tumoral function in papillary thyroid cancer (PTC) (53). In co-culture of PTC cells with primary monocytes or macrophage-like THP1 celles, high levels of CXCL16 were detected compared to separate PTC cell culture. Treatment of PTC cells with CXCL16 or co-culture with macrophages enhanced their migration potential. In turn, co-culture up-regulated the expression of M2-markers in macrophages, e.g., CD163, IL-10 and CD206, that was abrogated by an anti-CXCL16 antibody (53). An analysis of the TCGA PTC revealed an association of CXCL16 with M2 macrophage- and angiogenesis-related genes. High CXCL16 expression was associated with aggressive pathologic phenotypes, the higher TNM staging and lymph node metastasis in 77 patients with papillary thyroid cancers, in 25 patients with thyroid follicular adenomas, and 81 - with normal thyroid tissues from the SNUH cohort (51).

CXCL16 pro-tumoral activity was suggested for glioblastoma (GBM) patient’s (52). CXCL16 expression in GBM tissues was upregulated, compared to normal brain tissues. However, isolated tumor cells, even if cultured for 1-3 passages, had a substantial reduction in the CXCL16 expression levels. Treatment of mouse glioblastoma microglia with both recombinant and glioma-released CXCL16 increased the expression of anti-inflammatory genes ARG1, CHIL3, RETNLA and CD163 that was impaired by anti-CXCL16 antibodies. Microglia from glioma-bearing CXCR6-ko mice had lower expression levels of anti-inflammatory genes, compared to glioma-bearing wt mice that suggested CXCL16/CXCR6 axis involvement in the anti-inflammatory programming of microglia (**Figure 4**). Patient-derived GBM cells significantly increased cell chemotactic index, invasion and proliferation under CXCL16 exposure. Use of TCGA data with GBM patients revealed a significant increase in patient’s survival associated with CXCR6 deletion and a significant decrease in the survival associated with CXCR6 mRNA overexpression (52).

Human ovarian cancer tissue significantly increased expression of CXCL16 in comparison with both corresponding adjacent and para-cancerous tissues (54). The correlation analysis indicated a positive association of CXCL16 expression with an activation of macrophages in ovarian cancer (54). Macrophage-derived CXCL16 promoted migration and invasion of ovarian cancer SCOV3 cells by enhancing the activity of the PI3K/Akt pathway (54). Silencing of CXCR6 by shRNA in SCOV3 cells diminished above-mentioned effects of CXCL16 (54). In the co-culture of AIF1-overexpressed macrophages with hepatocellular carcinoma Hepa1-6 cells, CXCL16 secreted by macrophages enhanced proliferation and migration of cancer cells, and this effect was abrogated by a neutralizing antibody against CXCL16 (118).

We were able to find only one report describing CXCL16-mediating tumor-inhibiting function in colorectal cancer (CRC) (50). In co-culture of colorectal cancer SL4 cells with RAW 264.7 cells, CXCL16 induced tumor cell apoptosis mediated by TNFα-expressing macrophages. A susceptibility of CXCL16-overexpressing CRC cells to apoptosis was attenuated by neutralization of TNFα with a corresponding antibody (50).

In summary, CXCL16 tend to have predominantly a pro-tumoral role through promoting an anti-inflammatory phenotype of TAMs, and by activating proliferative and invasive potentials of cancer cells. Nevertheless, there is an evidence of CXCL16 anti-tumoral role too through sensitizing of CRC cells to apoptosis.

### 2.6 Class J scavenger receptors

#### 2.6.1 SR-J1/RAGE

SR-J1 (AGER, or RAGE) is a cell surface receptor from the immunoglobulin superfamily that specifically binds advanced glycation end products (AGEs) (119). RAGE is the only member of class J scavenger receptors and capable of binding multiple ligands (72). Except for AGEs, SR-J1 recognizes HMGB1 (120), members of the S100 protein family (121), amyloid-beta peptide (122) and binds dsDNA and dsRNA directly (123). RAGE is expressed by diverse cell types, including macrophages, monocytes, endothelial cells, fibroblasts and smooth muscle cells (124).

Multiple evidences indicate a pro-inflammatory role of RAGE activation, in particular, the HMGB1-induced activation of RAGE in inflammation-related context (125–127). RAGE is involved in ROS production and M1 polarization of macrophages under AGE exposure (65, 128). In macrophages, AGEs significantly elevated the expression of IL-6, IL-12, TNFα and TLR4, as well as the phosphorylation levels of STAT1 in cytoplasm in RAGE/ROS dependent manner (**Figure 4**). TLR4 inhibition by siRNA diminished effect of the AGE-dependent RAGE activation, while both RAGE expression and ROS production remained unchanged. This evidence suggests TLR4 as a downstream regulator of RAGE activation and further ROS production (65).

RAGE was studied in human GBM treated with temozolomide (TMZ) (129). TMZ treatment caused HMGB1 release from GBM cells in tumor tissue of patients. Affinity examination showed that RAGE is the main receptor binding extracellular HMGB1. Immunofluorescent analysis of patients` GBM samples indicated co-localization of RAGE and HMGB1 on TAMs. *In vitro* stimulation of THP-1 macrophages with recombinant HMGB1 promoted release of pro-inflammatory cytokines through NLRP3-dependent inflammasomes that was diminished by RAGE inhibition (129). The mechanism of RAGE activation by HMGB1 was related to phosphorylation of ERK1/2 and IKB resulting in NFκB activation. In patients with GBM, HMGB1 expression is associated with improved OS. These results indicate that RAGE interaction with HMGB1 can be favorable factor in GBM treatment response (129). Irreversible electroporation caused the release of nucleus HMGB1 out of PDAC cells followed by binding of HMGB1 to RAGE in THP1-derived macrophages, that skewed macrophages toward pro-inflammatory phenotype *via* MAPK-ERK activation (130). Macrophages enhanced phagocytosis of dying electroporated PDAC cells. This effect was neutralized by RAGE inhibition. MAPK-ERK inhibition significantly decreased the RAGE expression and the release of autocrine HMGB1 by macrophages (130).

*In vitro* RAGE is equally expressed in both M1- and M2-polarized macrophages, but has distinct effects on the cancer cells that depends on a polarization state of macrophages (69). In contrast to M1 macrophages, HMGB1-dependent stimulation of RAGE facilitated pro-tumor activity in M2 macrophages. RAGE activation by HMGB1 enhanced invasion of gastric tumor cells (MKN45) in co-culture with M2-polarized THP1 macrophages and vice versa with M1 macrophages (66). RAGE induced VEGF production in M2 macrophages. The conditioned medium of M2 macrophages treated with HMGB1 stimulated the growth of endothelial cells *in vitro*; this effect was opposite for M1 macrophages. In contrast to M1 polarization, the RAGE activation in M2 macrophages did not lead to NFkB activation. Two negative regulators of the NFkB activation, SOCS1 and SHIP-1, were significantly upregulated under the HMGB1 exposure in M2 macrophages (66). HMGB1-mediated RAGE activation in THP1-derived M2 macrophages also stimulated lymphangiogenesis by increasing both proliferation and migration of lymphatic endothelial cells as well as VEGF-C production in M2, but not in M0 macrophages (67). RAGE inhibition significantly reduced M2-dependent lymphangiogenesis (67). HMGB1+CD163+ M2 macrophages were found as detrimental prognostic factors for OS in laryngeal squamous cell carcinoma patients (67). RAGE mediates chemotaxis of THP1-differentiated macrophages upon stimulation with a conditioned medium of S100A7-overexpressing breast cancer MDA-MB-231 cells. This effect was significantly abrogated by RAGE blockage (131).

*In vivo* RAGE-depleted mouse models of GBM indicated RAGE as a significant TAM-specific factor participating in inflammation and angiogenesis in the TME (69). Survival analysis of tumor-bearing mice revealed that RAGE ablation significantly prolonged survival of mice in comparison with wild type (wt) mice. RAGE-depleted tumor exhibited lower expression of pro-inflammatory cytokines, and RAGE-depleted TAMs expressed significantly lower levels of IL-6 and VEGF-A. RAGE expression in tumor microglia or bone marrow-derived macrophages stimulated angiogenesis in GBM. Patient’s GBM samples had abundance of CD163+ TAMs with high RAGE expression (69).

RAGE was shown to mediate uptake of an oncogenic mutant KRASG12D protein by peripheral blood mononuclear cell-derived macrophages during autophagy-dependent ferroptosis of PDAC cells (68). Under oxidative stress conditions, tumor cells released KRASG12D protein *via* exosomes secretion. Exosomes were engulfed by macrophages in a RAGE-dependent manner that was confirmed by the knockdown of RAGE by shRNAs in macrophages. KRASG12D promoted M2 polarization *via* STAT3-dependent fatty acid oxidation (FAO). Inhibition of FAO reduced mRNA expression of IL10, ARG1, and TGFB1 in the macrophages. The knockdown of RAGE and ablation of STAT3 by shRNA abrogated the FAO and the M2 polarization. In PDAC patients, high KRASG12D expression in CD68+ cells correlated with worse OS rates. The KRASG12D uptake by macrophages may significantly contribute to the human PDAC progression (68).

In summary, RAGE activation of TAMs has controversial impact on TME and tumor cells. Evidence indicates that pro-tumor and anti-tumor RAGE role through distinct TAMs activation depends on TME context and RAGE ligands. Is of great interest to identify in future the spectrum of tumor-specific sets of RAGE ligands, and to examine how cooperation of M1 or M2-specific receptors with RAGE can a decide about pro-and anti-tumor programming of TAMs.

### 2.7 Class H scavenger receptors

The class H scavenger receptors are transmembrane protein receptors containing in their extracellular part fasciclin, EGF-like and lamin-type domains. Class H scavenger receptors has two members: SR-H1 (also known as Stabilin-1, or Clever-1) and SR-H2 (known as stabilin-2, or HARE) (132, 133). Despite high similarity in domain organization and endocytic functions, stabilin-1, but not stabilin-2 is expressed on TAMs and plays an essential role in tumor development.

#### 2.7.1 SR-H1/Stabilin-1

SR-H1, originally identified as stabilin-1 (134) and as CLEVER-1 (135) is multifunctional scavenger and intracellular sorting receptor with adhesive activities expressed by immunosuppressive monocytes and macrophages, sinusoidal endothelial cells and lymphatic endothelial cells (134, 136–138). Stabilin-1 performs endocytosis, phagocytosis, intracellular sorting of newly synthetized proteins and transcytosis of growth hormone family member placental lactogen (9, 139–144). Large body of evidence demonstrated that stabilin-1/CLEVER-1 can mediate cell-matrix and cell-cell interactions during primary tumor growth and in metastatic state (135, 138, 145–147).

Stabilin-1 is abundantly expressed on TAMs in number of solid cancers in patients and in murine models (8, 57, 132, 148). TAM-expressed stabilin-1 mediates clearance of tumor growth-inhibiting factor SPARC in a mouse model of breast cancer, and germinal knock-out of stabilin-1 results in the statistically significant reduction of primary tumor growth in this model (57). In orthotopic mouse models of lung cancer, breast cancer and lymphoma, genetic deficiency of macrophage stabilin-1 significantly reduced tumor growth (149). In stabilin-1 KO mice TME was shifted towards inflammatory program and was enriched in the activated endogenous CD8+ T cells. Immunotherapeutic blockade of stabilin-1 had similar consequences, and had synergistic effect with anti-PD-1 checkpoint inhibition (149).

Strong association of stabilin-1+ TAMs with worse prognosis was shown in several human cancers (55, 56, 150). Stabilin-1 expression in TAMs was associated with poor OS, RFS, tumor stage and histological grade in patients with urothelial carcinoma (56). High intratumoral expression of stabilin-1 on CD68+ TAMs was associated with poor DSS in stage I–IV rectal cancer (55). In contrast, high number of CD68+ stabilin-1+ TAMs correlated with longer DSS and predicted a favorable prognosis in early stage I colorectal cancer (CRC) patients (55).

Contribution of intracellular sorting function of stabilin-1 to tumor progression is linked to the ability of the extracellular domains of stabilin-1 to interact with at least two human chitinase-like proteins, SI-CLP and YKL-39, while the interaction with true chitinases CHIT1 and AMCase and with YKL-40 was not studied to date (6, 141, 151, 152).

We have demonstrated that stabilin-1 mediates intracellular delivery of newly synthetized SI-CLP, stabilin-1interacting chitinase-like protein, that interacted with a fasciclin domain of stabilin-1 in the yeast two-hybrid screening and in the affinity chromatography assay (141, 152). In a murine model for breast adenocarcinoma we demonstrated that SI-CLP being ectopically expressed in subcutaneously injected TS-A cells significantly reduced tumor growth and reduced infiltration of TAMs (153). Recently, we have also found that stabilin-1 is able to interact with YKL-39 (CHI3L2), that for a long-time was known as highly specific biomarker of rheumatoid arthritis, and later has been found to be overexpressed in glioblastoma affecting biology of transformed cells (6, 154). In patients with glioma high levels of CHI3L2 expressed in cancer cells and on microglia cells correlated with poor prognosis. Mechanistically the authors found that CHI3L2 induces the apoptosis of CD8+ T cells (155). We found that YKL-39 has two functions that can promote tumor growth: it stimulates monocyte migration, and it stimulates as well angiogenic activity of endothelial cells *in vitro* (6). In patients with breast cancer YKL-39 was exclusively expressed in TAMs in tumor mass, and elevated levels of YKL-39 in primary tumors significantly correlated with metastatic relapse after therapy onset (6). However, whether similar mechanisms can act in other types of cancer has to be studied, while application of purified SI-CLP and blocking agents for YKL-39 is a promising strategy to reprogram tumor-promoting microenvironment.

In summary, stabilin-1 has a highly complex function in cancer. Its deficiency is cancer-inhibiting, at least due to the reduction of SPARC clearance. Its ability to modulate concentrations of tumor-promoting YKL-39 and tumor-inhibiting SI-CLP can contribute to tumor growth and metastasis in a cancer-specific way, since not necessarily both proteins are present at the same time in TME. In particular, the role of stabilin-1 in CRC is of interest, while total amount of TAMs in this cancer type, in contrast to majority of other types, correlates with reduced tumor growth and good prognosis (8).

### 2.8 Class I scavenger receptors

SR-I (also known as CD163) is a hemoglobin-haptoglobin complex scavenger receptor that is mostly expressed in monocytes and macrophages (13, 156). In tumors, CD163 promotes tumor development and is associated with worth prognosis in breast cancer, head and neck cancer, lymphoma and melanoma (39, 59–62, 64, 100). CD163, mediates clearance of hemoglobin-haptoglobin complexes out of circulation is a silent way, however, in hyperglyceamic conditions this scavenging process leads to inflammatory macrophage responses (157).

CD163 is commonly defined as a marker for tumor-supporting TAM phenotype. In human breast cancer, CD163+ TAMs accumulation was inhibited by tumor suppressor TAp73 (58). Amount of CD163+ TAMs negatively correlated with Tap73 expression and positively correlated with tumor grade (58). High amount of CD68+ and CD163+ TAMs was associated with lymph node metastasis, high Ki67 expression and poor prognosis in 1579 breast cancer patients from Zhejiang Provincial People’s Hospital and Zhejiang Tiantai People’s Hospital (62, 100). Elevated levels of CD163+ TAMs in tumor stroma and tumor nest correlated with poor prognosis in 107 patients with triple negative breast cancer operated on at Dokkyo Medical University Hospital (59). CD163 was identified as a good predictor of pre-metastatic status of colorectal cancer (158). High levels of CD163+ cells were associated with tumor node metastasis stage, depth of infiltration, and lymphatic metastasis in 197 patients with colorectal cancer from China (158). Using multispectral immunofluorescence it was demonstrated that CD163+ cells have immunosuppressive phenotype in 17 patients with colorectal cancer who underwent resection of primary and liver metastases (159). High number of CD163+ cells was found in peritumoral region of tumor and in liver metastases (159).

Several studies confirmed that tumor-supporting effect of CD163+ TAMs is mediated by the activation of STAT3 signaling pathway (60, 63). Tumor-mediated activation of STAT3 in CD163+ TAMs resulted in pro-tumor TAM polarization (63). *In vitro*, conditioned medium from cholangiocarcinoma cell lines (HuCCT1, RBE and MEC) induced activation of STAT3 in modeled TAMs and enhanced production of IL-10, VEGF-α, TGF-β and MMP-2 in CD163+ TAMs (63) (**Figure 4**). CD163+ TAMs produced tumor-supporting cytokines (IL-6 and CXCL2) activated STAT3 in tumor cells and supported tumor progression. CD163-KO TAMs had decreased production of IL-6 and CXCL2 in comparison to WT TAMs in co-culture with MCA205 (mouse fibrosarcoma) cells (64). Conditioned medium of CD163-KO TAMs significantly impaired activation of STAT3 in MCA205 cells (64). CD163-expressing TAMs displayed elevated levels of pSTAT3 and correlated to poor prognosis in 77 patients with myeloma from STAT3 is over-activated within CD163pos bone marrow macrophages in both Multiple Myeloma and the benign pre-condition MGUS (60). Increased infiltration of both CD68+ and CD163+ TAMs in tumor mass correlated with decreased survival of 174 patients with gastroesophageal adenocarcinoma from Sweden (61). CD68 and CD163 overexpression was indicative for worse prognosis in 105 HCC patients from Japan (160). Increased levels of CD163+ TAMs correlated with decreased OS and higher histological grade in human sarcoma (64). Oppositely, in human primary melanoma low amount of CD163-expressing TAMs in tumor stroma was associated with recurrence and poor OS (39).

Overall, at least two molecular mechanism for tumor-supporting function of CD163+ TAMs were identified to date: inhibition of tumor suppressor TAp73 in breast cancer and activation of STAT3 signaling in TAMs and in r fibrosarcoma cells (58, 63, 64). Moreover, high expression of CD163+ TAMs was related to poor prognosis in breast cancer, gastroesophageal adenocarcinoma, HCC, human sarcoma, but not in melanoma patients (39, 59, 61, 62, 64).

## 3 Genetics of scavenger receptors

Deleterious germline mutations cause a broad range of distinct pathological conditions including cancer (161) There is limited information describing association of SR gene mutations with tumor progression, especially in non-malignant cells, e.g., macrophages. The only reliable evidence for such association are germline mutations in MSR1 coding scavenger receptor CD204 (162, 163). Genetic analysis of hereditary prostate cancer revealed significant co-segregation of prostate cancer with the nonsense mutation R293X in man of European descent and the missense mutation D174Y in man of African American descent (163). The truncating mutation R293X resulted in deletion of most of the collagen-like domain of MSR1 gene, including the ligand-binding region and the cysteine-rich domain. The missense mutation D174Y can affect proper polymerization of three MSR1 polypeptide chains. Both mutations disrupted MSR1 function that affected MSR1 ability to bind oxLDL involved in the oxidative stress. MSR1 is predominantly expressed by macrophages in both benign and cancerous prostate tissues, emphasizing the role of macrophage-derived mutated MSR1 in prostate cancer development (163).

MSR1 mutations are also involved in Barrett esophagus (BE) and esophageal adenocarcinoma (EAC) development (162). The nonsense R293X and missense L254V mutations contributed to BE/EAC risk, or were required for BE/EAC predisposition. The L254V mutation was found within the conserved coiled-coil domain of MSR1, so both R293X and L254V led to MSR1 function disruption. MSR1 mutation caused overexpression of key nuclear cell cycle molecule Cyclin D1 (CCND1) in BE and EAC tissue samples that was impaired by overexpression of wild-type MSR1 in HEK293 cells (162).

Association of MSR1 mutations with progression of prostate cancer and esophageal adenocarcinoma confirmed the involvement of this scavenger receptor to carcionogenesis.

## 4 Conclusions

Macrophage SRs have dual role in tumor development. Tumor-supporting activity mediated by macrophage SRs includes regulation of tumor invasion, proliferation and migration (for CD204, CD206, CXCL16, Stabilin-1, and RAGE), as well as M2-like TAM polarization (for CD36, LOX-1, CXCL16, CD 163, and RAGE) and tumor angiogenesis (for CD68, Dectin-1, RAGE). The anti-tumor functions of TAM-expressing SRs include suppression of tumor angiogenesis (for CD204), tumor invasion (for RAGE), inducing tumor cells clearance (for MARCO) and M1-like TAM polarization (for CD204 and RAGE). In cancer patients, number of TAM-expressed SRs (CD204, MARCO, CD68, LOX-1, Dectin-1, CD206, CXCL16, Stabilin-1, CD163, and RAGE) associates with negative and more sever prognosis. Targeting of tumor-promoting SRs can be a promising approach in cancer immunotherapy. Accumulating clinical data demonstrate that SRs can serve as potential prognostic biomarkers for patients with cancer (29, 84, 164). For example, in mouse model of triple negative breast cancer specific targeting of CD206+ TAMs inhibited tumorigenesis and metastatic dissemination of tumor cells (165). Application of antibodies against MARCO resulted in the reduction of tumor growth and inhibition of metastasis in murine models for melanoma and breast cancer (166). There are multiple studies describing effects of SR targeting in various cellular *in vitro* and pre-clinical *in vivo* models (145, 165, 167–169). Drug targeting of CD36 demonstrated promising results for patients with advanced soft tissue sarcoma in the initial clinical trials (167, 170). However, targeting CD36 failed in phase 2 clinical trials because of ineffective performance and severe adverse events. The complications can be explained by the expression of majority of various SRs on resident macrophages and on other cell types in healthy organs and tissues. Moreover, we still have very limited information about cancer-specific ligands of SRs, in particular for the ability of SRs to internalize and target for degradation cytokines and growth factors.

The investigation of the mechanisms of tumor development and progression mediated by SRs is a foreground goal for the developing immunotherapeutic approaches that can help to suppress tumor cell invasion, proliferation and migration, to inhibit macrophage recruitment and pro-tumor macrophage polarization as well as to enhance clearance of tumor cells by TAMs. Moreover, the ability of SRs to internalize both particles and molecular complexes still remains to be explored for the design of targeted drug delivery for macrophage re-programming in tumor microenvironment.

## Author contributions

EK performed literature analysis and drafted the manuscript. EK designed Figures. PI and IL contributed to literature analysis and wrote manuscript chapters. JK developed the concept of the manuscript, designed tables, wrote manuscript chapters, edited final text. All authors contributed to manuscript revision, read and approved the submitted version.
